# Advances in Healthcare Human Factors and Informatics: Contributions from the 2024 Literature

**DOI:** 10.1055/s-0046-1816626

**Published:** 2026-11-03

**Authors:** Vignesh Subbian, Anthony Solomonides

**Affiliations:** 1 The University of Arizona, Tucson, AZUSA; 2 Research Institute, Endeavor Health, Evanston, ILUSA

**Keywords:** Human Factors, Medical Informatics, Sociotechnical Issues, Digital Health

## Abstract

**Objective** : To summarize significant research contributions published in 2024 related to human factors and organizational issues (HFOI) in healthcare and to select notable papers for the HFOI section of the International Medical Informatics Association (IMIA) Yearbook 2025.

**Methods** : We refreshed a previous search query for identifying HFOI research and executed the query in PubMed in January 2025. Papers were evaluated based on methodological rigor, contribution to understanding human-technology interactions in healthcare settings. The resulting articles were sent for external review to identity best paper candidates.

**Results** : We retrieved 1,939 articles related to HFOI, of which six articles were selected as candidates for best papers. The general themes from the best paper candidates include (1) applying user-centered design as a foundational principle, (2) optimizing data visualizations and information recall, (3) navigating complex clinical workflows, and (4) the role of interdisciplinary collaborations.

**Conclusion** : Human factors in health informatics continues to evolve toward more sophisticated, theory-driven approaches that integrate multiple disciplinary perspectives while maintaining practical applicability for real-world healthcare settings.

## 1. Introduction

This manuscript represents the synopsis for the human factors and organizational issues (HFOI) section of the 2025 International Medical Informatics Association (IMIA) Yearbook. The aim of this synopsis is to summarize significant research in the HFOI domain and to select the best papers published in this field in 2024. Building on decades of foundational work in multiple disciplines related to health informatics, researchers have increasingly recognized that successful health technology implementation requires careful consideration of human factors at multiple levels—from individual user interactions to complex organizational and policy contexts [ 1 2 ]. The 2024 literature demonstrates growing recognition that healthcare systems are complex sociotechnical environments where human performance emerges from dynamic interactions between people, technology, tasks, and organizational contexts. As artificial intelligence and digital health technologies proliferate, understanding these interactions becomes increasingly critical for ensuring safe, effective, and sustainable implementations. This synopsis supplements the review paper on HFOI and planetary health, authored by Tyagi *et al.* [ 3 ]. The papers reviewed here represent diverse methodological approaches, from rigorous application of established human factors frameworks to innovative theoretical developments that expand our understanding of human-technology interactions in healthcare.

## 2. Methods

We used an updated search strategy from the HFOI synopsis in the 2024 IMIA Yearbook [ 2 ]. We are including the actual query in Table 1 to support future Yearbook contributors. Similar to the prior synopsis, we searched for relevant papers in PubMed® using the revised search strategy. We included journal articles published in 2024 in English and had a series of keywords either in the MeSH terms or in the paper's title or abstract, with particular attention to papers addressing “human factors,” “user-centered design,” and “sociotechnical systems,” in healthcare contexts. The final query is shown in Table 1 .

**Table 1.**
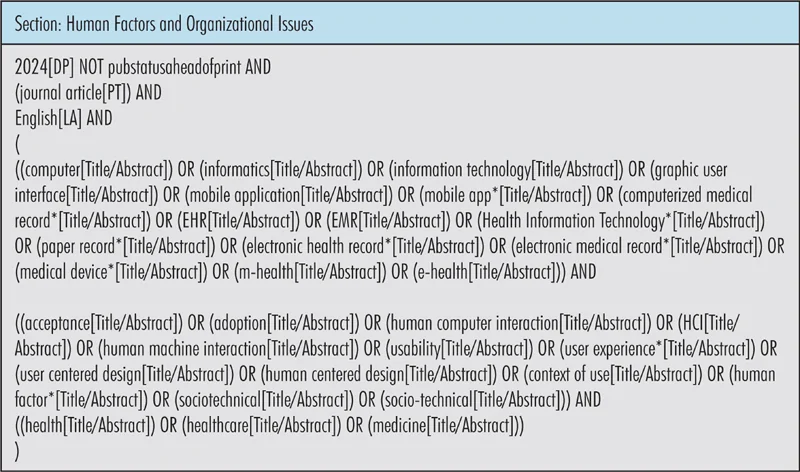
Search Strategy for HFOI Literature Review.

The selection process involved multiple stages of review, beginning with title and abstract screening followed by full-text evaluation. Papers were assessed for methodological rigor, theoretical contribution, and practical significance. Special consideration was given to studies demonstrating novel approaches to longstanding clinical and/or sociotechnical challenges in healthcare and those providing exemplary applications of established methodologies. The full IMIA Yearbook editorial board determined the best paper finalists and honorable mentions.

## 3. Results

The search yielded 1,939 unique references. Following title and abstract screening, 437 references were retained for full-text review. Of these, nine were selected as best paper candidates. After external evaluation, the IMIA Yearbook editorial committee identified one paper as the 2024 Best Paper in HFOI and five papers as honorable mentions ( Table 2 ). Briefly, these include: a User-Centered Framework for Implementation of Technology (UFIT) such as clinical decision support (CDS) systems [ 4 ], flu vaccination dashboards [ 5 ], a mobile health app for tobacco cessation called Sense2Quit [ 6 ], research-design collaborations [ 7 ], a pediatric electronic Patient-Reported Outcome Measure (ePROM) Platform called Voxe [ 8 ], and evaluation of sepsis care transitions [ 9 ]. A summary of these six best paper candidates can be found in the appendix of this synopsis.

**Table 2.**
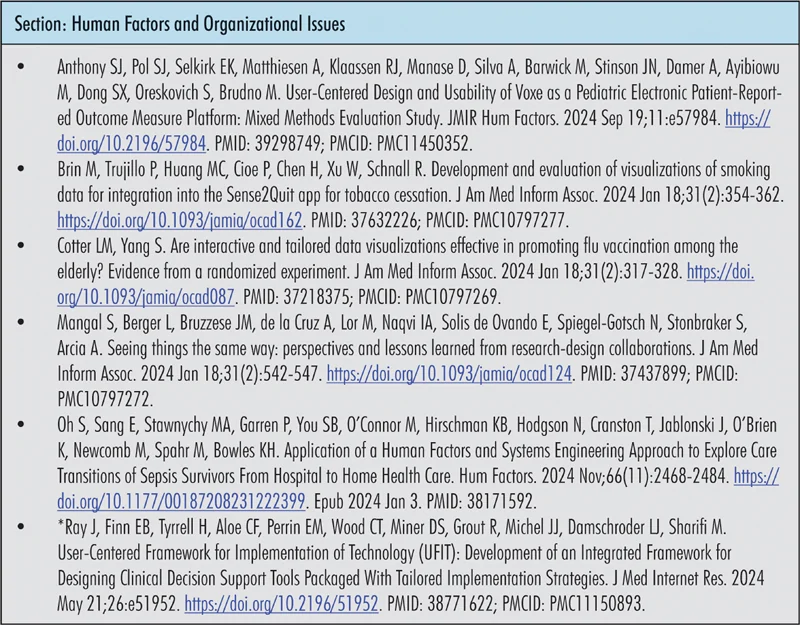
Selection of best papers for the 2025 IMIA Yearbook of Medical Informatics for the section Human Factors and Organizational Issues. The articles are listed in alphabetical order of the first author's last name. *Denotes special consideration and best paper finalist.

### 3.1. User-Centered Design as a Foundational Principle

A dominant theme across the six studies is the role of user-centered design (UCD) in creating, implementing and evaluating effective health technologies. Studies demonstrate that involving end-users (patients, caregivers, and healthcare providers) and experts (informaticians, designers) throughout the design and evaluation processes is fundamental to success. The development of the Sense2Quit app for persons living with HIV to support tobacco cessation exemplifies this, with 66 changes made across five rounds of usability testing based on feedback from both end-users and informaticians [ 6 ]. Similarly, the Voxe pediatric ePROM platform underwent iterative testing with children and healthcare providers, leading to high satisfaction and demonstrated user-friendliness [ 8 ]. UCD allowed for tailoring of features to particular populations, such as Voxe's child-centered personalization options (colors, avatars, GIFs) that increased motivation and engagement for pediatric patients. The UFIT framework explicitly integrates UCD principles with human factors and implementation science, emphasizing that UCD should not only guide technology creation but also the design of tailored implementation strategies that promote adoption in diverse clinical settings [ 4 ].

### 3.2. Optimizing Data Visualizations and Information Recall

The effectiveness of data visualizations in health communication was another common thematic research area. While visualizations are widely used to convey health information, their impact on behavior and recall is complex. The study on flu vaccination dashboards found that while dashboards generally increased perceived susceptibility to flu, interactivity did not improve vaccination intentions or information recall, and could even decrease recall, particularly among older adults [ 5 ]. This challenges the common assumption that more interactivity is always better, suggesting it can introduce respondent burden or distraction. In contrast, the flu vaccination study strongly supported the benefit of explanatory text alongside visualizations and demonstrated increased information recall, especially for the elderly population. For the Sense2Quit app, visualizations of smoking behaviors (e.g., cash spent, daily trends) were refined to be interpretable and actionable and allow for self-management of cessation efforts [ 6 ].

### 3.3. Navigating Complex Clinical Workflows and Systemic Barriers

The selected studies continue to show that healthcare environments are intricate sociotechnical systems where technology implementation must account for existing workflows, human factors, and organizational structures. For example, a critical challenge identified in the sepsis care transition study is the “information transfer deficit” from hospitals to home health care. This information loss, often due to sepsis diagnosis being marked as “resolved” before discharge, can prevent clinicians from providing appropriate aftercare and increase readmission risks. The study highlighted that transitional care processes often face more barriers ( *e.g.* , patient refusal, staffing issues, lack of primary care physicians) than facilitators compared to hospital processes [ 9 ]. Similarly, the UFIT framework highlights the need to tailor CDS tools and implementation strategies to diverse clinical settings by thoroughly analyzing existing electronic health record (EHR) use patterns and workflow variations. The study recognized that clinicians have different ways of interacting with EHRs during patient visits ( *e.g.* , clustered, limited, or integrated use), necessitating a multi-pronged design approach [ 4 ]. Both UFIT and the sepsis care transition study explicitly use human factors approaches ( *e.g.* , systems engineering initiative for patient safety – SEIPS framework) to map complex care processes, identify work system elements (persons, tasks, tools, organization, environment), and pinpoint barriers and facilitators. Such deep understanding of the “local context” is shown to be critical for successful implementation of health information technologies [ 10 11 ].

### 3.4. The Imperative of Interdisciplinary Collaboration

Successful health informatics innovations are rarely the product of a single discipline; they necessitate close collaboration among researchers, designers, health information professionals, and other domain experts [ 12 ]. The paper on research-design collaborations emphasizes that researchers bring data understanding, while designers apply aesthetic and usability principles [ 7 ]. This synergy is important for high-quality visual products. For example, in the Sense2Quit app development, discrepancies between end-user feedback and expert heuristic evaluations were common, demonstrating that integrating both perspectives, along with design expertise, was essential for a usable and visually appealing mobile health application [ 6 ].

## 4. Conclusion

The insights from the selected HFOI studies underscore that developing impactful digital health solutions requires a holistic, user-centered approach that extends beyond initial technology development to effectively understand and integrate with existing human behaviors, clinical workflows, and organizational contexts. While innovative features such as interactivity show promise, their effectiveness must be carefully evaluated for specific populations, and often, simpler design choices such as clear explanatory text prove more beneficial. More importantly, the success of these initiatives relies heavily on robust interdisciplinary collaboration, fostering mutual understanding and leveraging diverse expertise to overcome inherent challenges in information transfer, workflow integration, and user engagement.

### Acknowledgement

We are thankful Kate Fultz Hollis and Lina Soualmia for leading the 2025 IMIA Yearbook of Medical Informatics as well as the reviewers for their contribution to the selection process.
